# Fabrication and Optimization of Stable, Optically Transparent, and Reusable pH-Responsive Silk Membranes

**DOI:** 10.3390/ijms17111897

**Published:** 2016-11-15

**Authors:** Andreas Toytziaridis, Cedric Dicko

**Affiliations:** Pure and Applied Biochemistry, Chemical Center, Lund University, 22241 Lund, Sweden; andreas.toytziaridis.999@student.lu.se

**Keywords:** chromogenic, pH-responsive, silk patch, neutral red, thionine, Laponite, membrane

## Abstract

The fabrication of silk-based membranes that are stable, optically transparent and reusable is yet to be achieved. To address this bottleneck we have developed a method to produce transparent chromogenic silk patches that are optically responsive to pH. The patches were produced by blending regenerated silk fibroin (RSF), Laponite RD (nano clay) and the organic dyes neutral red and Thionine acetate. The Laponite RD played a central role in the patch mechanical integrity and prevention of dye leaching. The process was optimized using a factorial design to maximize the patch response to pH by UV absorbance and fluorescence emission. New patches of the optimized protocol, made from solutions containing 125 μM neutral red or 250 μM of Thionine and 15 mg/mL silk, were further tested for operational stability over several cycles of pH altering. Stability, performance, and reusability were achieved over the tested cycles. The approach could be extended to other reporting molecules or enzymes able to bind to Laponite.

## 1. Introduction

*Bombyx mori* silk can be broken down, solubilized and regenerated into different formats, either in water or in organic solvents in a series of simple processes [1]. These good properties have earned silk positions in several different areas outside the textile industry, such as tissue engineering, disease modeling, implant devices, drug delivery, and as immobilization matrix. The first report of enzyme immobilization was by Grasset et al. [2] where they successfully cross-linked aminotransferase to woven silk fabrics; and later on, immobilization/entrapment of enzymes in silk powders [3] and membranes followed [4]. For the last two formats, it was quickly recognized that leakage of the biomolecules was critical and correlated to the β-sheet content of the silk matrix.

The more flexible format of a silk membrane for immobilization (mainly of enzymes) has been explored further [5,6,7,8] with particular emphasis on the control of stabilization of biomacromolecules and minimal leakage [9,10]. The success of the methods did, however, result in a non-transparent membrane in most cases. It is only recently with the exploration of silk as corneal implants [11,12] and bioelectronics support matrix [13,14] that silk membrane displayed optical transparency, flexibility, and strength. The biomacromolecules to entrap still need to be large and the control of the β-sheet content requires delicate post-treatment.

To address those challenges and be able to entrap molecules of any size and reduce the number of processing steps, we propose to combine silk proteins and the synthetic clay Laponite RD.

Laponite, also known as hectorite, is a synthetic inorganic smectite clay, which has very specific dimensions. Its structure forms oval platelets, 25 nm long and about 1 nm thick. The structure is comprised of a sheet of mostly Mg^2+^ and some Li^+^. On each side of the metal ion sheet is a layer of silicon oxide. The flat faces of the platelet are negatively charged, while the thin edge of the nanoparticle with its hydroxyl groups has a net positive charge. The exchangeable cations capacity (CEC) is nominally 55 mEq/100 g (i.e., milliequivalent of hydrogen per 100 g of dry clay powder). Stacked Laponite can be exfoliated in water yielding a transparent dispersion. Below 2% (*w*/*v*) the dispersion is a liquid and above a gel.

The charged nature and the interlayer space of clays like Laponite have made them widely interesting for the adsorption of organic molecules. Many different molecules have been adsorbed onto Laponite, and by modifying the Laponite by cation exchange, more adsorption properties, such as greater hydrophobicity, can be obtained [15,16,17,18,19,20,21,22]. Intercalated dyes have been shown to gain increased stability and pigments based on nano-clays present great properties as colorants. Additionally, nanoclays can improve the structural properties of polymers [23,24,25], molecules insoluble in water can be dispersed by the help of Laponite [18], Laponite shows promise as a drug delivery carrier [26] and enzymes have been adsorbed onto clays for use as biocatalysts and biosensors [15]. To our knowledge, only two studies have explored the silk fibroin and nanoclays interactions to create materials with enhanced properties. One focused on montmorillonite [25] to produce nanocomposites and the other on Laponite XLG to produce injectable hydrogel [27]. In both cases, the optical transparency was not achieved and the reusability not discussed.

Can we then make an optically transparent silk membrane that is stable and reusable with a reporting mechanism? Focusing on optical-sensing, some recent work using quantum dots [28] and copper nanoparticles [29] in silk membrane for hydrogen peroxide and pH sensing respectively, demonstrated that sensing worked. In both cases however the membranes were neither fully transparent nor reusable.

We describe in this paper the process of manufacturing in water transparent, mechanically and operationally stable membranes which are optically responsive to pH. The films were made of regenerated silk fibroin (technical grade silk) from *Bombyx mori* entrapping nanoplatelets of Laponite onto which the organic pH indicator dyes neutral red or thionine acetate was adsorbed. This demonstrated the possibilities of using Laponite-doped regenerated silk to make functional biomaterials. As Laponite nanoparticles have versatile adsorption capabilities, many other functional molecules, such as enzymes, could be entrapped in the silk matrix to achieve similar properties.

## 2. Results

**Figure 1:**
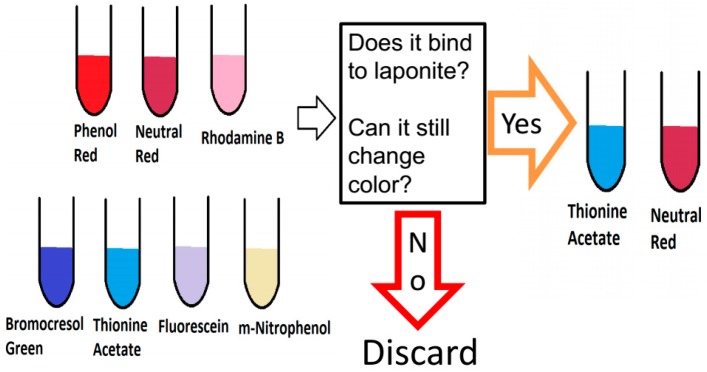
Summary of the procedure for the dye selection. We selected only the dyes showing irreversible binding to Laponite, and change of color upon pH change (visually, by UV-Vis absorption and fluorescence emission).

Seven organic dyes (neutral red, thionine acetate, bromocresol green, rhodamine B, phenol red, fluorescein, 3-nitrophenol) were screened with Laponite (LP) in solution (Figure 1). The clay–dye combinations were analyzed with a UV-Vis spectrophotometer and a spectrofluorimeter (data not shown) at different pH to see if they retained their pH-dependent color change and if they were bound irreversibly to the Laponite.

Among the seven different dyes, neutral red (NR) and Thionine acetate (Th) combined with Laponite were chosen as the best candidates for a silk film based optical pH-sensor. After, dye selection, we tested the non-optimized fabrication of a silk film containing Laponite and dye (see materials and methods). The critical parameters tested were transparency and homogeneity, since those properties are desirable when analyzing with optical instruments.

### 2.1. Spectral Properties of LP–NR, LP–Th and Non-Optimized Silk Films Modified with LP–NR and LP–Th

**Figure 2:**
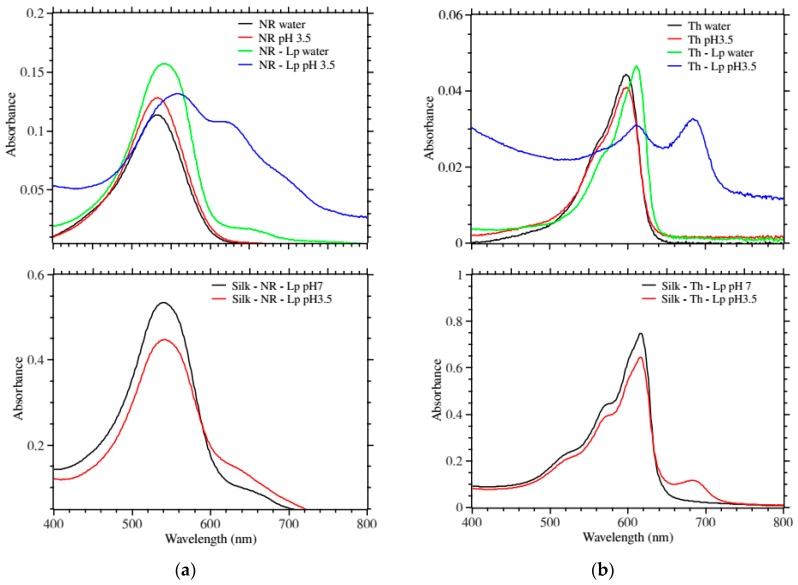
UV-Vis absorbance profiles. (**a**) **Top**, comparison of free neutral red in water, at pH 3.5; and, neutral red bound to Laponite in water and at pH 3.5. With acidification, the Laponite modified neutral red displayed new red peaks; **Bottom**, comparison of silk patches containing neutral red and Laponite at pH 7 and pH 3.5. The main peak intensity decreased and a new red peak appeared; (**b**) **Top**, comparison of free thionine in water and at pH 3.5; and thionine bound to Laponite in water and at pH 3.5. With acidification, the Laponite modified thionine displayed strong new red peaks; **Bottom**, comparison of silk patches containing thionine and Laponite at pH 7 and pH 3.5. The main peak intensity decreased and a new stronger red peak appeared.

Both dye-candidates displayed shifts of their main absorbance peak upon mixing with Laponite (Figure 2a,b top panels). This is a common phenomenon seen when molecules are placed in environments of different polarity, which in turn can be taken as an indication that the dyes were bound to the Laponite. Interestingly, this also introduced new peaks at higher wavelengths (640 nm for neutral red and 680 nm for thionine) at pH 3.5 for both dyes when they were mixed with Laponite compared to only buffer (Figure 2a,b top panels).

This is a phenomenon seen with dyes that form multimer at high concentration through π–π interactions and is called metachromasy [30]. The appearance and increase of the new peaks were chosen as the most remarkable signal of pH change.

Figure 2a,b show the UV-Vis absorbance response of the silk–dye–Laponite in water and at pH 3.5. Similarly to the dye–Laponite, we observed a decrease of the main peak upon acidification and the appearance and increase of a new peak. Th showed a stronger effect.

**Figure 3:**
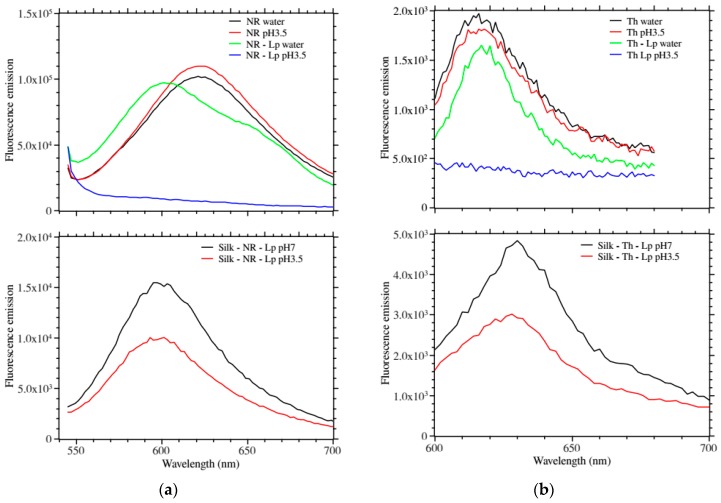
Fluorescence emission profiles. (**a**) **Top**, comparison of free neutral red in water, at pH 3.5; and neutral red bound to Laponite in water and at pH 3.5 (excitation at 540 nm). With acidification, the Laponite modified neutral red displayed a strong quenching as compared to the free dye; **Bottom**, comparison of silk patches containing neutral red and Laponite at pH 7 and pH 3.5. The fluorescence emission was quenched at low pH for both dyes; (**b**) **Top**, comparison of free thionine in water and at pH 3.5; and thionine bound to Laponite in water and at pH 3.5 (excitation at 595 nm). With acidification, the Laponite modified thionine displayed strong quenching as compared to the free dye; **Bottom**, comparison of silk patches containing thionine and Laponite at pH 7 and pH 3.5. The fluorescence emission was quenched at low pH for both dyes.

Figure 3a,b show the fluorescence emission of the above films. In solution, only the LP–NR showed a shifted fluorescence emission as compared to the free dye. Remarkably, when lowering the pH to 3.5 both LP–NR and LP–Th fluorescence emissions were completely quenched (Figure 3a,b top panels).

Figure 3a,b bottom panels show the fluorescence emission of the silk–dye–Laponite in water and at pH 3.5. We observed a measurable quenching of the fluorescence emission at lower pH.

Results from Figure 2 and Figure 3 reinforced our choice of NR and Th as multi-modal reporting dyes for our silk-based pH-sensor. The key to the success of these dyes probably lies in the fact that they are always cationic (and attracted to the anionic Laponite) and that they have a flat aromatic structure that could also bind to the oxygen layer of the Laponite by π-stacking [16,31]. It was observed during the experiments that the absorbance peaks for NR shifted 5–10 nm over night or over a couple of days. Previous studies have ascribed this behavior to formations of dye multimer and/or the dye transitioning from the exterior to the interior of the clay [31]. It was decided that the dye should be left to mature with the Laponite before mixing with the silk with the motivation that the dye transition should go faster in solution than in the silk films and that the films should be as stable as possible in terms of absorbance before use.

During the preliminary experiments (results not shown) it was also observed that Laponite slowly increases the pH of its surrounding solution towards about pH 9, which is the pH of freshly prepared Laponite solution. Even Laponite immobilized in silk films had this effect. A film freshly made and rehydrated, but not washed, would typically increase pH from 5.5 to 6.5 over 30 min. This effect is a problem because the pH-responsive film should not affect its environment and thus affect its own response. This is why an extensive washing period of 22 h in 200 mL + 200 mL deionized water was included in the protocol. The pH-changing effect of the Laponite was decreased to about 0.05–0.1 pH-units over 30 min. Most remarkably, with this extensive washing the silk patch remained stable (visual inspection and silk protein peak by UV absorbance) and the dye did not leach. This suggested a tight network of the Laponite–dye–silk structures.

### 2.2. Preparation and Pre-Conditioning of Silk Patch

**Figure 4:**
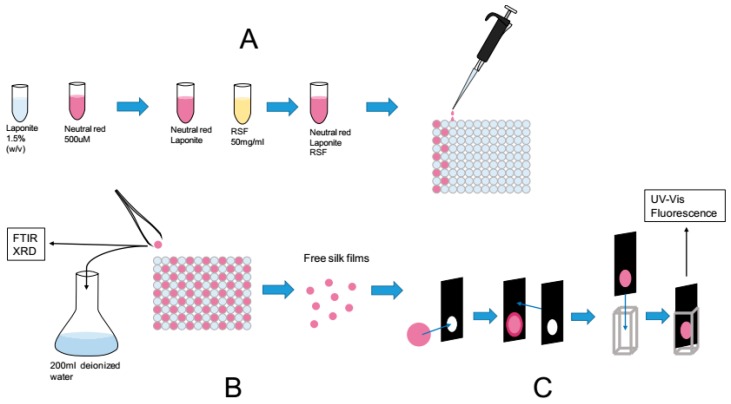
Summary of procedures for the fabrication and characterization of chromogenic silk patches: (**A**) Mixing and casting of RSF/Laponite/Dye solution; (**B**) After overnight drying the ready patch were tested by FTIR and XRD; and, immersed in 200 mL deionized water to test the dye leakage; (**C**) Custom built holder for the silk patch fitting in a 1 cm standard cuvette. The chosen geometry allows UV absorbance and fluorescence spectra to be collected in solution and without disruption of the sample. Scale: the silk patches are standardized to a diameter of 8 mm.

Following our pre-optimization studies we designed a workflow to reproducibly manufacture silk films modified with Laponite and dyes. Figure 4 illustrates the three main steps. Note that the dye concentrations were kept below the cation exchange capacity of the Laponite RD (i.e., 55 mEq/100 g).

### 2.3. Factorial Design of Silk Patch Fabrication and Optimization

To systematically explore the interplay between silk, Laponite and dye we designed a full factorial experiment (see materials and methods). The Laponite concentration was kept constant to a final concentration of 0.375% (*w*/*v*). Table 1 summarizes the factors used, their range and the sample list.

Note that samples 19, 20 and 21 were controls and not part of the factorial design; they were added to the table for convenience.

The samples were prepared according to the scheme in Figure 4. Not all films were recoverable from the 96 well plate lids. The samples containing the lowest amount of silk were not recoverable. These were only analyzed by FTIR but excluded from the factorial design analysis.

**Figure 5:**
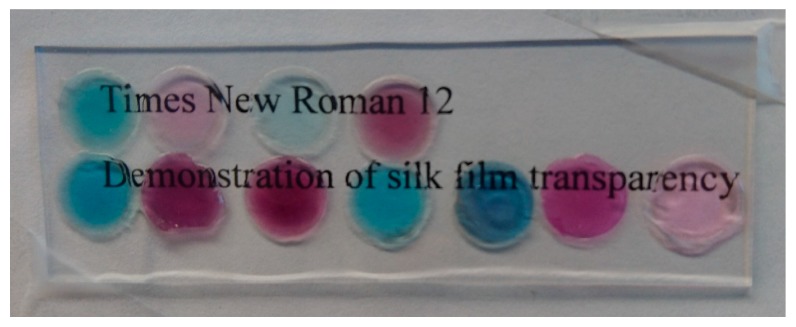
Photograph of selected silk–dye–patches (red for neutral red and blue for thionine) on a glass slide. Behind the patches and the glass slides are lines of text to illustrate the transparency of the patches. For convenience, the patches were wetted to adhere on the glass slide.

Figure 5 demonstrates the transparency of the obtained silk patches. Here a selected amount of silk patch containing either NR (red) or Th (blue) were placed on a microscope glass slide and behind were lines of text clearly visible. Note that for convenience the patches were wetted to adhere on the glass slide.

Each sample from the factorial design was then measured as follows: (i) FTIR characterization (Amide I/II ratio and crystallinity); (ii) XRD characterization; (iii) dye leakage estimate by UV absorbance; (iii) UV absorbance change upon acidification from pH 7 to pH 3.5; (iv) maximum fluorescence emission change upon acidification from pH 7 to pH 3.5.

**Figure 6:**
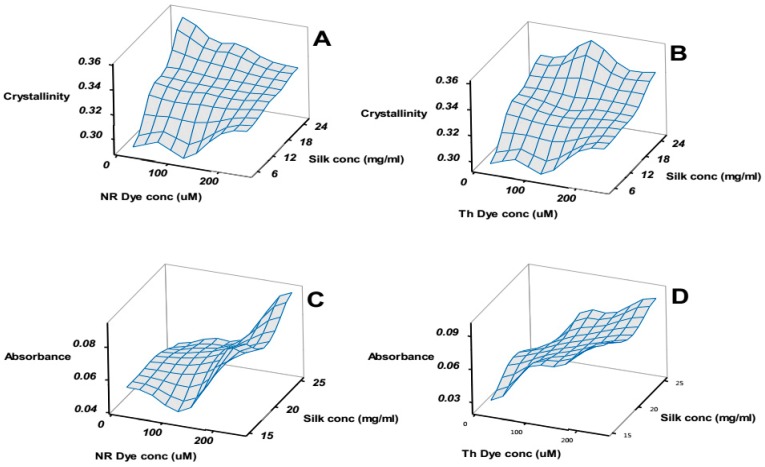
3D mesh plots of selected responses as a function of factors in factorial design. Panels (**A**,**B**) compare the patch crystallinity (see materials and methods) for the NR and Th patches respectively; Panels (**C**,**D**) compare the increase of absorbance from the new peak in NR and Th patches respectively. The success of the patch hinged on a compromise between crystallinity (mechanical integrity) and high absorbance (optimal pH response).

Before the statistical evaluation, some qualitative results can be highlighted. Figure 6A,B compare the change in the FTIR calculated crystallinity (see materials and methods) in each of the samples as a function of silk concentration and dye concentration for NR and Th respectively. Figure 6C,D compares the change in absorbance observed in the new peak as a function of silk concentration and dye concentration.

Figure 6, in essence, compares the structural aspects (A and B) to the functional aspects (C and D) of the silk patches. A naïve observation was that maximizing the functional response did not correlate to a stronger patch. To clarify the interplay and optimize the patch we conducted the analysis of the factorial design on the amide I/II ratio, crystallinity, change in UV absorbance (at main peak and at the new peak) and change in maximum fluorescence emission. The amide I/II ratio was sensitive to global structural changes in the silk patch. The crystallinity provided a more precise measure of the “stiffness” [32].

Table 2 summarizes the statistical analysis of the factorial design (analysis of variance and regression). Briefly, for each observable, the most significant factors and their combination are listed with their *p*-value. The most significant factors are marked in bold (i.e., with a *p*-value < 0.05). The *F*-value helps to compare the importance of the factors relatively to one another. The higher the *F*-value the more important is the factor. The correlation coefficients *R*^2^ and *R*^2^ prediction provide an estimate of how well the model explains the data and how well the model can predict new data respectively. Finally, the model *p*-value summarizes the significance of the correlation coefficient.

The structural observables Amide I/II ratio (global change in secondary structure) and crystallinity both depended on silk concentration (see Figure S1 for details), which meant the silk/Laponite ratio. A higher silk/Laponite ratio meant higher crystallinity and lower amide I/II ratio. The dye concentration also seemed to affect the structural properties, but to a less predictable degree.

The best-fit relationship between the factors and the functional observables was the increase in absorbance of the new peak (640 nm for NR and 680 nm for Th). Of all the significant factors, the dye concentration had the largest *F*-value. Interestingly, the model significance and high predicted *R*^2^ for this observable suggested a good reliability.

The decreases in absorbance of the main peak absorbance or the fluorescence were not fitted with significance. For the fluorescence, the quality of the measurements could also explain the marginal effect observed in the *p*-values.

Using the models from the factorial design analysis, we further exploited our results using a multiple response optimization with a maximization constraint for the functional observation (i.e., highest response possible). We found, that the optimal patch to maximize the optical responses to pH change was sample 9 (see Table 1) made from solution containing 125 μM NR with 15 mg/mL silk. Sample 16 (see Table 1) made from solution containing 250 μM Th with 15 mg/mL silk was a close enough optimum to be also considered.

#### 2.3.1. X-ray Powder Diffraction

The samples with highest silk concentration (in Table 1), films of Laponite-silk and Laponite-dye were analyzed by XRD to estimate the Laponite stacking and possible intercalation of the dye in the clay interlayer spacing. The diffractograms (Figure S2) showed no expected peak at 6–7 degrees of diffraction angle, which is associated with the interlayer space distance [33]. This indicated that the Laponite was completely exfoliated and consequently the dyes would be on the clays surfaces. No detectable silk β-peak was visible. This may be explained by the detection limit of the instrument.

#### 2.3.2. Dye Leakage

To evaluate the dye leakage from the films in Table 1, one of each film was placed in 1 mL milliQ water and incubated in a shake incubated, 180 rpm at room temperature for 84 h. The absorbance spectra and fluorescence spectra of the wash water were recorded and compared to the spectra of solutions containing the same amount of dye as the sample film (see Figures S3 and S4). None of the samples showed a measurable leakage.

#### 2.3.3. Photostability

The optimum patch (sample 9) was tested for photostability (see materials and methods). Exposure to an intense halogen lamp showed no significant differences in photostability between the Laponite–NR in solution (Figure S5) and the silk–Laponite–NR patch in solution (Figure S6).

### 2.4. Operational Stability

The optimal film (sample 9) and the next best (sample 16) were subjected to alternating pH for multiple cycles to assess the operational stability of the absorbance and fluorescence responses. This was performed by measuring the absorbance and fluorescence emission spectra each time after the film was placed into a different pH buffer.

**Figure 7:**
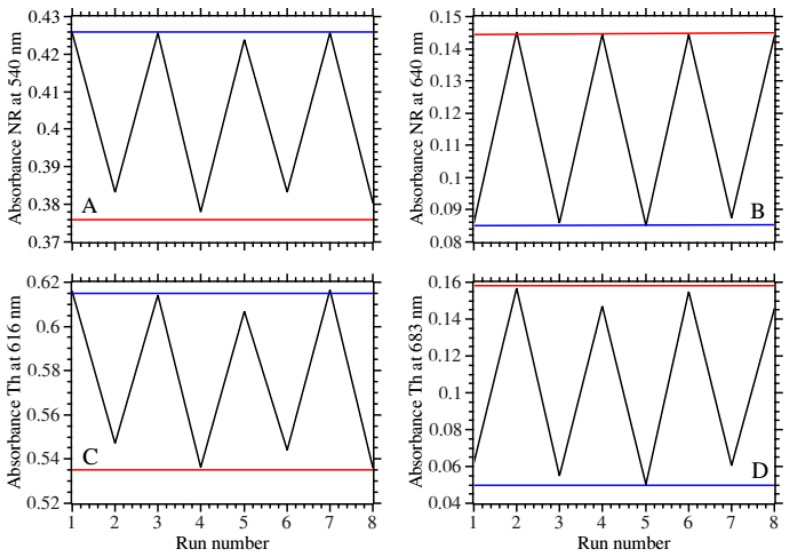
Operational stability: successive monitoring of absorbance and fluorescence in pH 7 and pH 3.5. Panels (**A**,**B**): neutral red patch; Panels (**C**,**D**): Thionine patch. In all panels the blue line corresponds to the absorbance pH 7 (every odd run number); the red line corresponds to the absorbance at pH 3.5 (every even run number).

Figure 7A,B shows the change in the main peak absorbance and the new peak for NR. Figure 7C,D shows the same for Th. For both type of patches the responses oscillated reproducibly from maximum to minimum values for the 8 cycles tested. The response amplitude was larger for the new peak absorbance for both types of patches. The fluorescence spectra showed less consistency (Figure S7). This was attributed to the stability of the measuring setup.

## 3. Discussion

The most remarkable aspect of the results is that we can achieve good optical transparency, limited to non-existent dye leakage and operational stability, all at the same time. The simplicity of the process, water-based mixing of stock solutions, is the key to understanding the mechanism underlying the membrane final properties.

**Figure 8:**
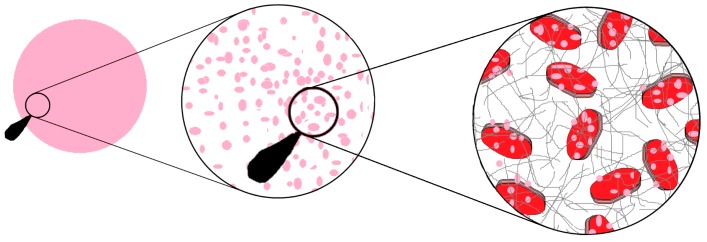
Possible structural organization of a pH-responsive silk patch. Represented are the silk proteins, the Laponite platelets and on the Laponite platelets the dye molecules.

Figure 8 illustrates the putative organization of the different components for the silk patch.

The first determining factor here was the pH of the Laponite stock solution, which was approximately 9. Laponite and hectorite clays are Lewis bases [34]. Even after addition of the dye the pH remained alkaline at 9 pH units. When adding the RSF the pH was lowered to 7 but slowly drifted back to pH 9. The buffering capacity of the Laponite drove the silk to a non-aggregating state, far from the dimerization pH of the termini domains and gelling points [35,36]. Consequently, an extensive washing of the film was needed to reduce the Laponite buffering capacity. Surprisingly, the extensive washing did not affect significantly the silk secondary structure [32]. It appears that the structural composition of the patch is solely decided by the silk–Laponite ratio. This is in sharp contrast with Dang et al. [25] who used acidic condition in a silk/montmorillonite resulting in an overlapping effect of silk acidic fibrillation and electrostatic interactions with the clay. A more complex picture of the fine interplay and balance between protein structure and processes is detailed by Hamman et al. [37].

A second determining factor, not explored here, was the organization and distribution of the Laponite in the silk film [38]. Pawar et al. [39] found for example in a Laponite—gelatin system that local reorganization of the gelatin by the Laponite could impart new and enhanced mechanical properties.

In a recent contribution Zink et al. [40] review systematically the transformation of biopolymers to produce films and coating. The focus on bioplastics and existing industrial processing methods highlights the progress and challenges so far, but may not provide a suitable route for sensing solution and biofunctional properties.

In conclusion, we demonstrated the necessary steps and interactions for using Laponite-doped regenerated silk to make stable, pH-sensitive and reusable silk membranes. The process is simple and allows for technical grade components in water. Potentially, using Laponite nanoparticles versatile adsorption capabilities, many other functional molecules [16,18,26,41,42], and enzymes [15,43], could be entrapped in the silk membrane.

## 4. Materials and Methods

### 4.1. Materials

Soap degummed *Bombyx mori* silk was obtained from an online store. Lithium bromide and neutral red were obtained from Sigma-Aldrich (Steinheim, Germany). Thionine acetate was obtained from Merck chemicals (Darmstadt, Germany). Laponite RD was a gift from Jon-Otto Fossum and sourced from BYK Additives and Instruments (Widnes, UK).

### 4.2. Silk Regeneration

7.8 g Lithium bromide was dissolved in 10 mL deionized water at 70 °C. 1.1 g of degummed silk was added and dissolved with the help of mechanical mixing (glass rod). When the mix was homogeneous in terms of viscosity (there were no lumps) the solution was incubated 15 min at 70 °C and was stirred at regular intervals with a glass rod. The solution was then filtered through a piece of sterile 100% cotton gauze pads (5 cm × 5 cm pads from local pharmacy), used as a sieve to remove debris from the regeneration process. Approximately 15 mL of filtered solution was then dialyzed in a 12 kDa cellulose tube for 3 days against 5 L deionized water. The dialysis water was switched every 10 h. After dialysis, the regenerated silk fibroin (RSF) was transferred into a 50 mL falcon tube, centrifuged 30 min at 1500 relative centrifugal force (RCF) and stored in a refrigerator at 8 °C a day before use.

### 4.3. Preparation of Silk Films Containing Clay–Dye Hybrids for Dye Screening

The dye stocks were prepared at 30 mM and the Laponite stock at 1.5% *w*/*v*. The Laponite-dye stocks were prepared in a 1:1 ratio and left to equilibrate at least 24 h in a refrigerator at 8 °C. The final solution of silk–dye–Laponite was a 1:1:1 mix of Laponite–dye hybrid stock, RSF at a concentration of approximately 50 mg/mL and water. The mix was gently agitated then aliquoted to 100 μL at a time onto the inside of a 96-well plate lid, where the rims for each well held the droplet in a circular shape with a diameter of 8 mm. The droplets were then left to dry 17 h over night at 25 °C and 35% relative humidity, on the laboratory bench. The formed patches were rehydrated in deionized water before use.

### 4.4. Preparation of Silk Films Containing Clay–Dye Hybrids for Factorial Design

A Laponite stock was prepared by mixing Laponite RD with milliQ water over night and under stirring (300 rpm) to a concentration of 1.5% (*w*/*v*). Thionine acetate and neutral red were used directly to prepare stock solutions in milliQ water. All solutions were stored at 8 °C in a refrigerator. The procedure follows:

Step 1: the Laponite stock 1.5% (*w*/*v*) was mixed with Thionine acetate or neutral red stocks of concentrations 1, 0.5 and 0.1 mM to a ratio of 1:1 to make 6 different Laponite–dye stocks. The Laponite–dye stocks were left in refrigerator overnight to mature.

Step 2: each Laponite–dye stock was mixed with RSF of 50, 30 and 10 mg/mL concentration to a 1:1 volume ratio. Mixing was kept to a minimum to avoid shearing and inducing fibrillation of the solution. The final concentration of Laponite in each sample was 0.375% (*w*/*v*) and the final concentrations of the other components can be seen in Table 1 in the results section, where three control samples are also included. The controls were prepared with un-dyed Laponite. The samples were designed using a full factorial design (Minitab17 software, Minitab Inc., www.Minitab.com, State College, PA, USA).

Step 3: the mix (silk–Laponite–dye) was then portioned out 100 μL at a time onto the inside of a lid of a 96-well plate, where the rims for each well held the droplet in a circular shape with a diameter of 8 mm. The droplets were then left to dry over night at room temperature on the laboratory bench, under mild air circulation. For each sample, 5 patches were cast.

Step 4: the formed films were rehydrated and washed by incubation with 200 mL deionized water for 18 h and then incubated for 4 more hours with 200 mL fresh deionized water. Both incubations were in shake flasks in a cell culture incubator rotating at 180 RPM at room temperature.

### 4.5. pH Measurement

The pH was measured using a Hamilton HI2210 pH-meter equipped with a Hamilton MiniTrode and temperature sensor (Hamilton, Bonaduz, Switzerland).

### 4.6. Dye Leaching Test

To quantify the dyes leakage, one new patch of each sample from Table 1, was incubated in 1 mL of deionized water for 84 h in a shake incubator at room temperature and at 180 rpm. The absorbance-spectra and fluorescence spectra excited at 540 nm for neutral red and 595 nm for thionine of the 1 mL incubation water were recorded. The control for this test was 1 mL of water containing the same amount of dye that the sample film did contain nominally. For example, the control for a sample film made with 125 μM neutral red was 1 mL water containing 12.5 μM neutral red. This corresponded to maximum leakage of dye and would be used to estimate the amount of dye leached.

### 4.7. Photostability Test

To assess the photostability of the patch, a selected film from factorial design optimization was placed in the cuvette holder using the cuvette setup described in Figure 4C. The cuvette was exposed to a 150 W halogen lamp placed 1 cm away and for 5 h. The lamp was a Fiber-lite MI-150 high intensity illuminator from Dolan Jenner Industries (color temperature 3250 K). The experiment was conducted in a cold room at 8 °C to rule out temperature effects. The patch was immersed in potassium phosphate buffer, 10 mM, and pH 7 and left at least 15 min to temperature equilibrate. Dye alone was run as a control. Absorbance and fluorescence spectra were recorded at every 30 min during the whole irradiation period.

### 4.8. UV-Vis Absorbance Characterization

Spectra were collected on a Cary 60 UV-Vis spectrophotometer from Agilent technologies, in the range 800–200 nm using the setup described in Figure 4C. The buffers used were potassium phosphate buffer, 10 mM, pH 7 and citric acid buffer, 10 mM, pH 3.5.

### 4.9. Fluorescence Emission Characterization

Fluorescence emission spectra excited at 540 nm for neutral red and 595 nm for thionine were recorded with a Quantamaster 40 spectrofluorimeter from PTI, using a special holder (Figure 4C). The buffers used were the same as for UV absorbance.

### 4.10. X-ray Powder Diffraction Characterization

The films with the highest concentration of silk were measured by X-ray diffraction (XRD) along with Laponite dye stocks, Laponite solution 1.5% and RSF 50 mg/mL, 0.1 mL each dried onto standard Mylar disks. The instrument used was a Stoe Stadi MP X-ray diffractometer equipped with a curved germanium monochromator and a MYTHEN detector (Darmstadt, Germany). Samples were collected in transmission geometry and scanned in the 2θ range of 2–90 degrees.

### 4.11. Fourier Transform Infrared Attenuated Total Reflectance (FTIR-ATR) Characterization

Each sample was analyzed on a Thermo Fisher iS5 FTIR instrument using a diamond ATR accessory (Thermo Fisher Scientific Inc., Waltham, MA, USA). 64 scans were co-added at 4 cm^−1^ resolution in the range 500–4000 cm^−1^. The crystallinity index [44] was estimated by fitting and computing the following equation:

(1) $$\mathrm{Crystallinity}=\frac{A_{1260}}{A_{1260}+A_{1230}}$$

where *A*_1260_ and *A*_1230_ are the FTIR-ATR absorbance at 1260 cm^−1^ and 1230 cm^−1^ respectively. Note that Laponite and dye do not absorb at those frequencies. The amide I/II ratio [45] was computed by first subtracting a linear baseline between covering the Amide I and II region; followed by computing the ratio of the peak maxima of the Amide I and II respectively.

### 4.12. Statistical Analysis

The factorial design and optimization were analyzed using Minitab17 software. Briefly, the factorial design was analyzed using analysis of variance (ANOVA) and regression analysis. The model obtained from the ANOVA and regression analysis was further used to predict the optimum process conditions using the Determinant-optimality (D-optimal) criterion. Specifically, Minitab minimizes the variance of the regression coefficients in the model by selecting design points that satisfy the D-optimal criterion from a set of candidate points.

## Figures and Tables

**Table 1:** Factorial design samples.

Sample Number ^1^	Run Number	Dye Concentration (mM)	Silk Concentration (mg/mL)	Dye Type
1	16	25	5	NR
2	6	25	5	Th
3	1	25	15	NR
4	7	25	15	Th
5	3	25	25	NR
6	8	25	25	Th
7	13	125	5	NR
8	14	125	5	Th
9	12	125	15	NR
10	11	125	15	Th
11	15	125	25	NR
12	4	125	25	Th
13	5	250	5	NR
14	17	250	5	Th
15	18	250	15	NR
16	9	250	15	Th
17	2	250	25	NR
18	10	250	25	Th
19	19	0	5	–
20	20	0	15	–
21	21	0	25	–

^1^ Sample 19, 20 and 21 are the dye free controls and not part of the factorial design.

**Table 2:** Analysis of variance and regression results.

Observable	Factors ^1^	Factor *p*-Value	*F*-Value	*R*^2^ (%)	*R*^2^ (%) Prediction	Model *p*-Value
Amide ratio	Silk	0.001	73.98	97.45	48.46	0.014
Crystallinity	Dye	0.067	5.7	98.39	67.35	0.006
	Silk	0.000	89.10			
	Dye + silk	0.015	12.7			
Decrease main peak absorbance	Dye	0.051	18.73	98.54	47.51	0.064
	Dye type	0.033	28.85			
	Dye + Dye type	0.056	16.74			
Increase new peak absorbance	Dye	0.001	1406.08	99.94	98	0.002
	Dye + silk	0.034	28.28			
	Dye + Dye type	0.003	350.91			
	Silk + Dye type	0.060	15.11			
Decrease of Fluorescence	Dye	0.045	21.22	98.13	32.56	0.082
	Dye type	0.028	33.97			

^1^ Dye: dye concentration; silk: silk concentration; dye type: neutral red or thionine.

## Supplementary Materials

Supplementary materials can be found at www.mdpi.com/1422-0067/17/11/1897/s1.

## Abbreviations

NR	Neutral red
RSF	Regenerated silk fibroin
Th	Thionine acetate
Lp	Laponite
