# Supplementary material for: Fabrication and Optimization of Stable, Optically Transparent, and Reusable pH-Responsive Silk Membranes
Source: Int J Mol Sci. 2016 Nov 15;17(11):1897. doi: 10.3390/ijms17111897 (PMC5133896; doi:10.3390/ijms17111897)
Supplement: Supplementary file 1 [file ijms-17-01897-s001.pdf]

Supplementary Materials: Fabrication and Optimization of Stable, Optically Transparent, and Reusable pH-Responsive Silk Membranes

Andreas Toytziaridis and Cedric Dicko

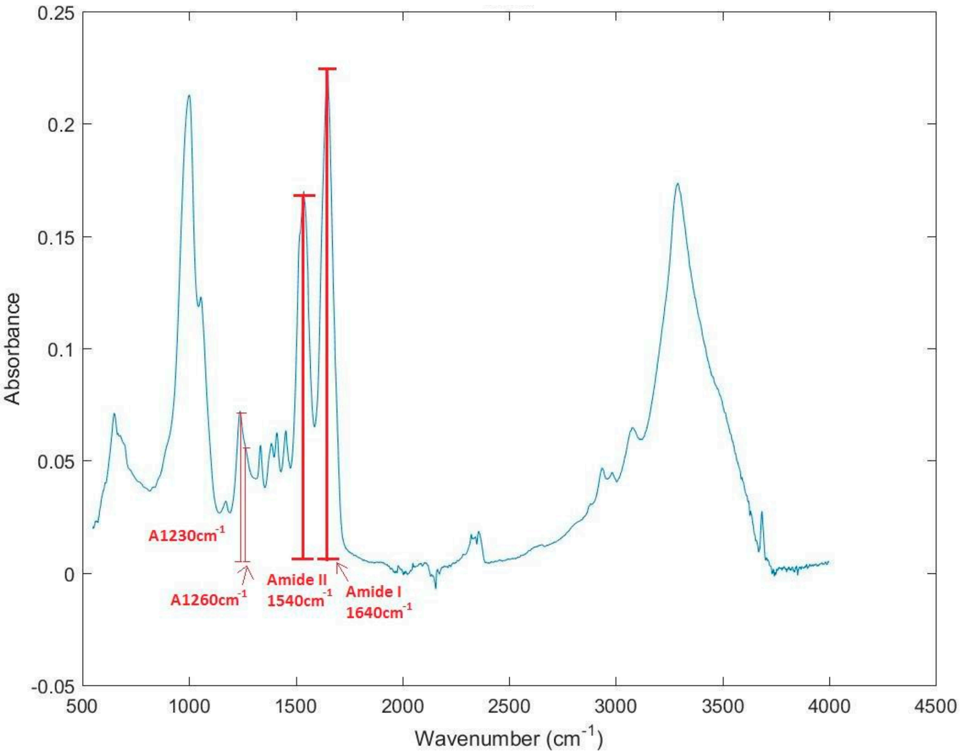

Figure S1. FTIR-ATR of sample 6 (see Table 1) silk-laponite-dye patch and regions of interest for crystallinity and amide I/II ratio.

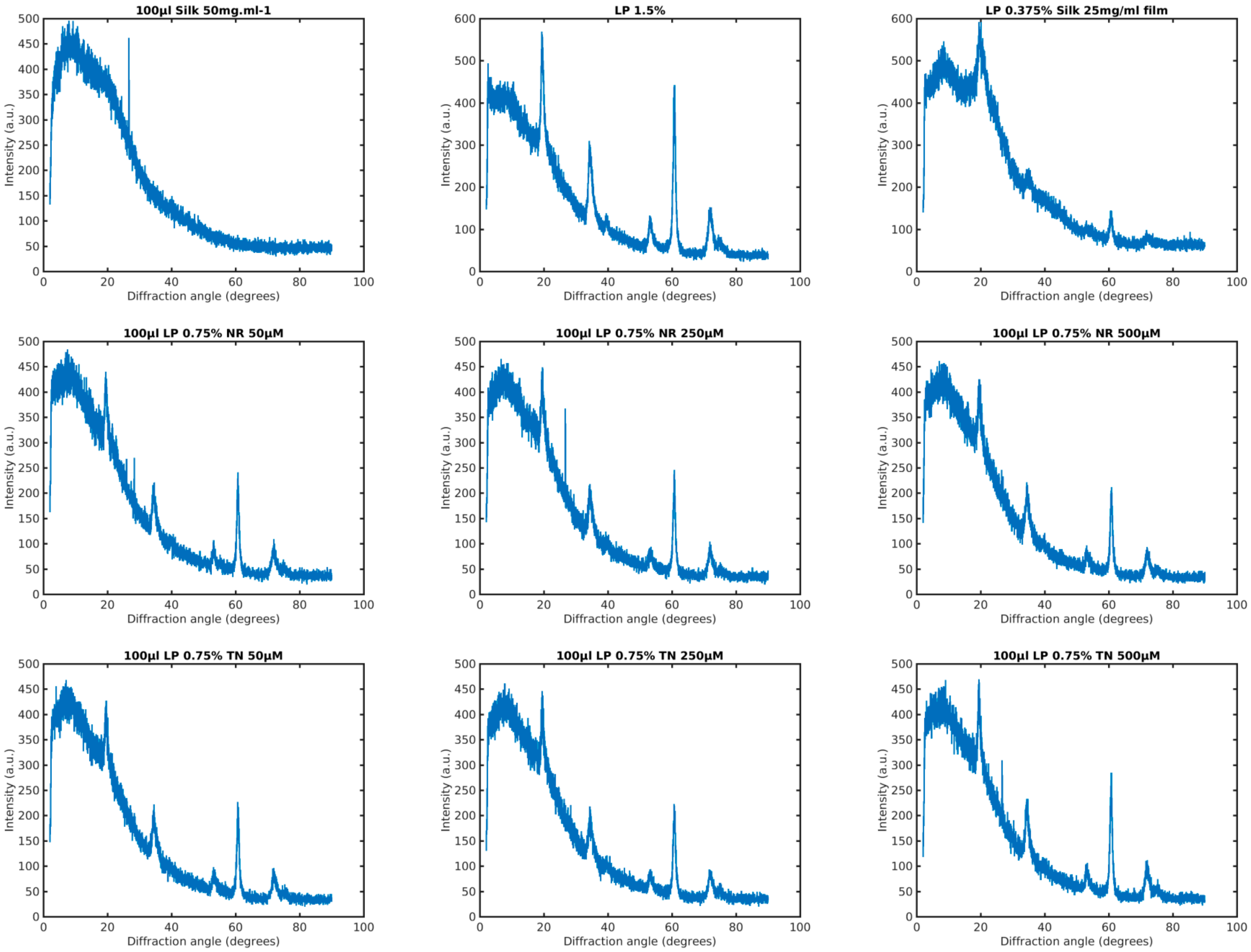
100µl Silk 50mg.ml-1
LP 1.5%
LP 0.375% Silk 25mg/ml film
100µl LP 0.75% NR 50µM
100µl LP 0.75% NR 250µM
100µl LP 0.75% NR 500µM
100µl LP 0.75% TN 50µM
100µl LP 0.75% TN 250µM
100µl LP 0.75% TN 500µM
Intensity (a.u.)
Diffraction angle (degrees)

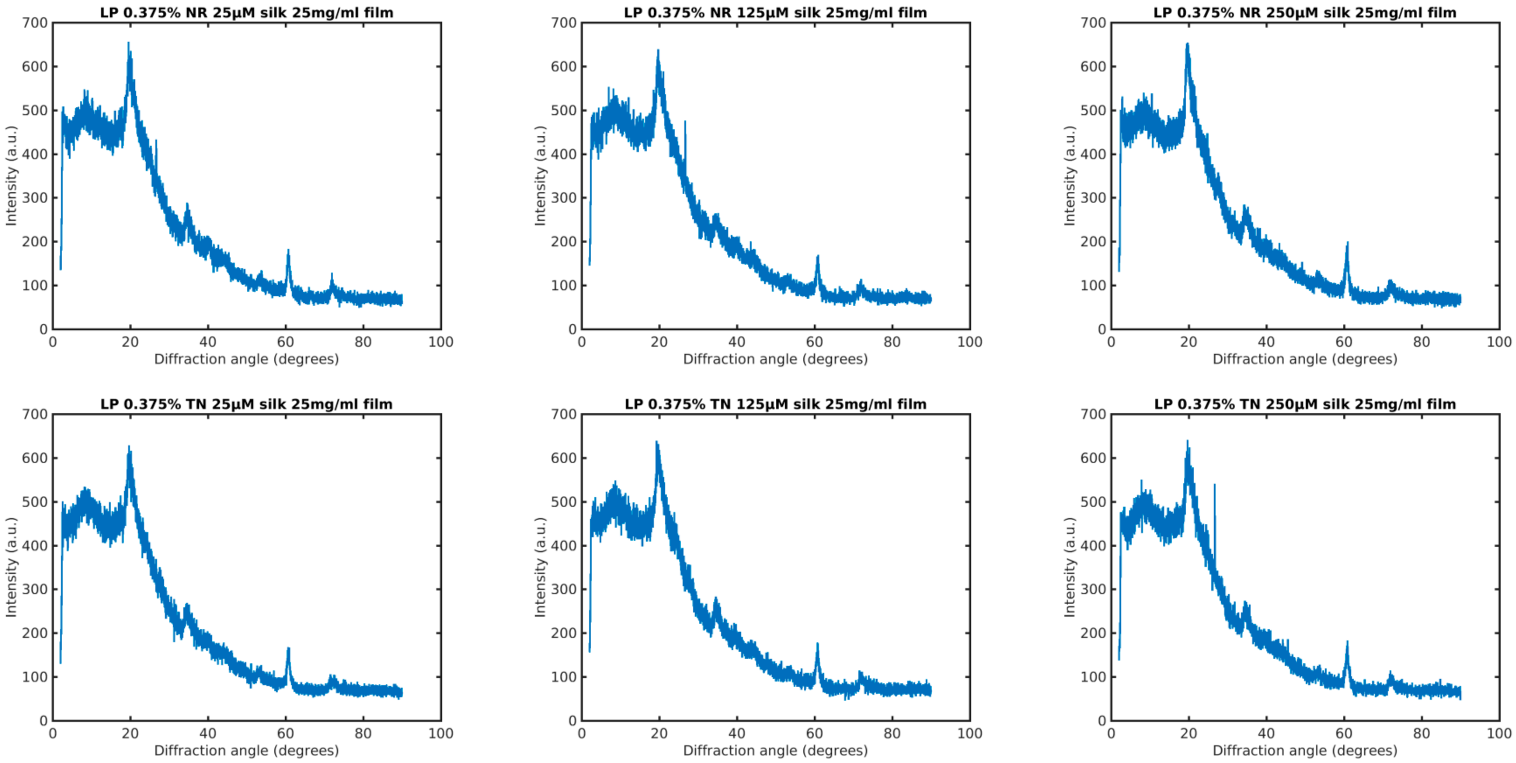

Figure S2. XRD diffractogram of samples from Table 1, except the 5 mg/mL silk patches.

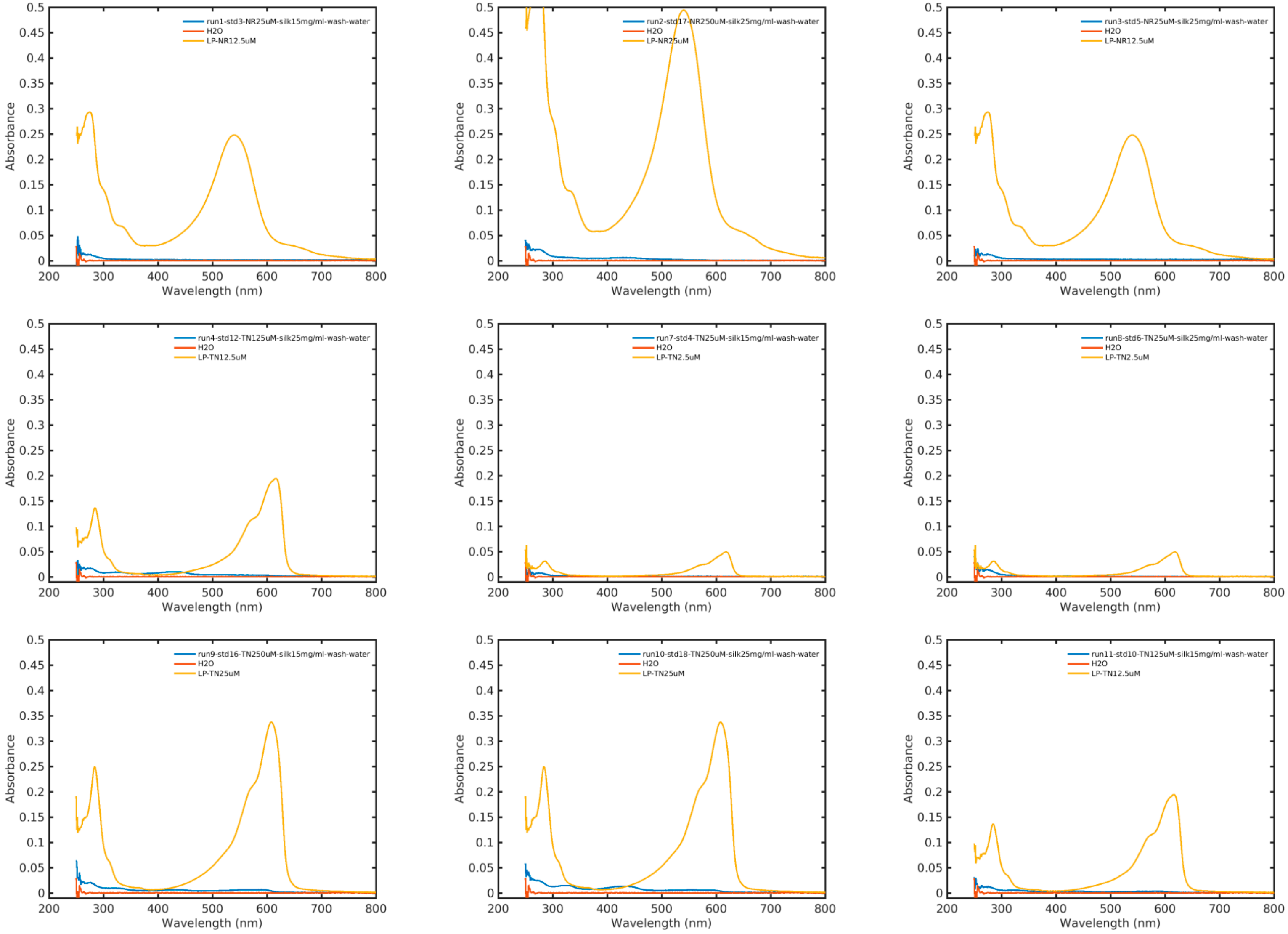

Figure S3. *Cont*.

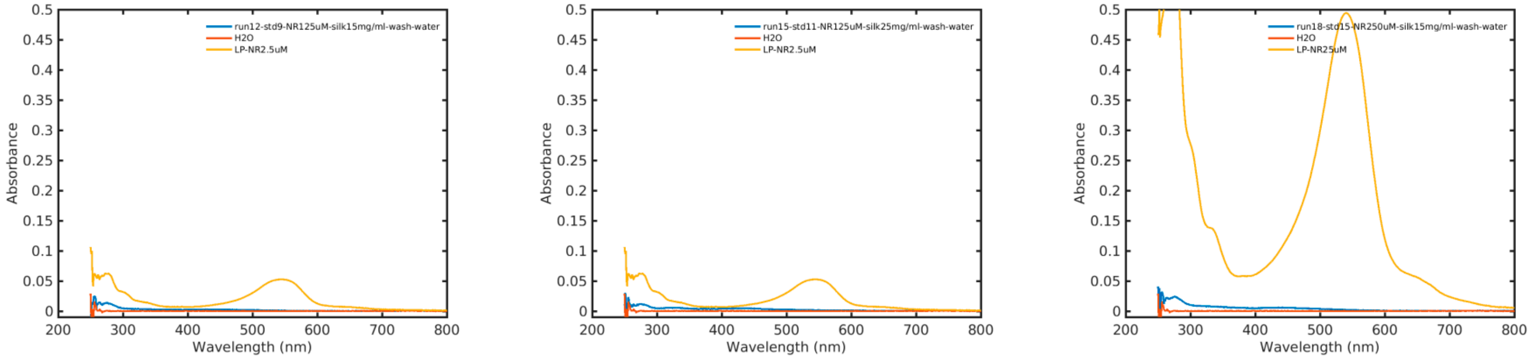

Figure S3. Leakage evaluation by absorbance.

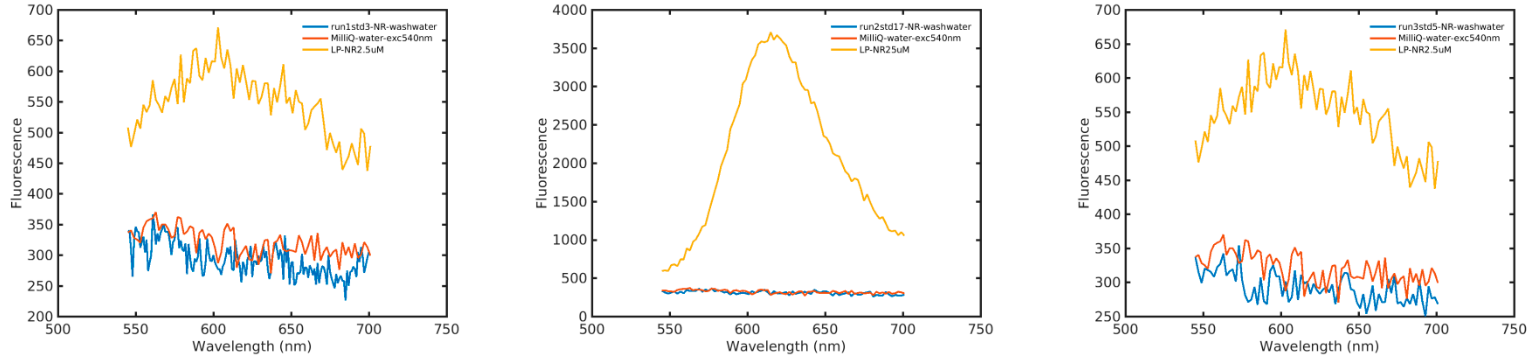

Figure S4. *Cont.*

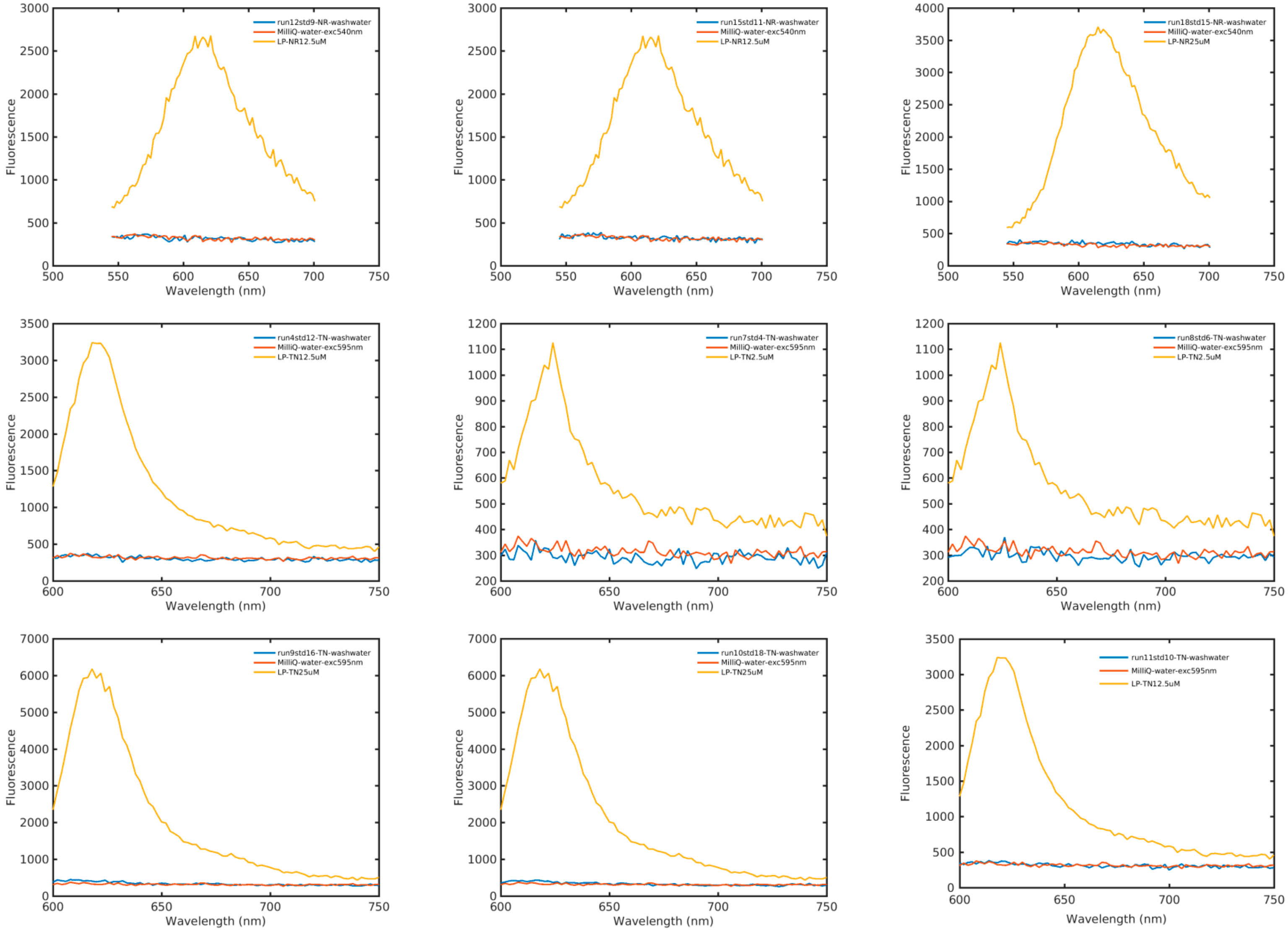

Figure S4. Leakage evaluation by fluorescence.

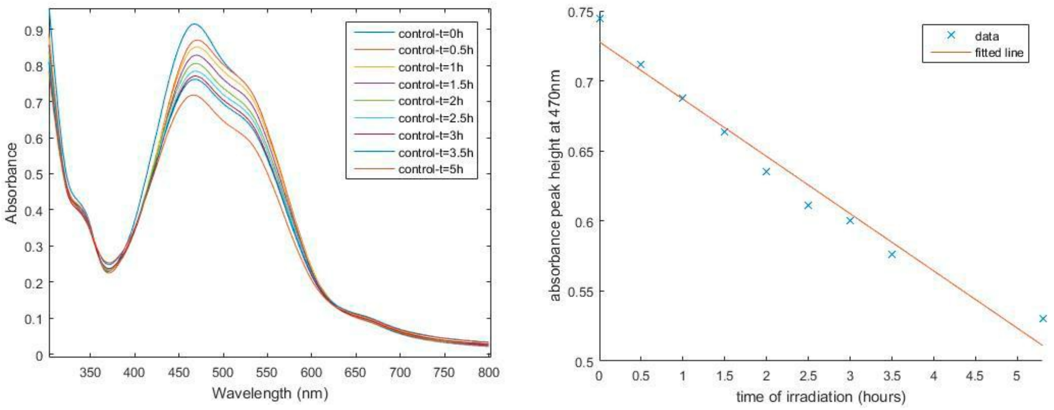

(**a**) (**b**)

Figure S5. Photostability test for NR: (**a**) Absorbance spectra of 1.5 mL potassium phosphate buffer, 10 mM, pH 7, with 125 μM Neutral red at different times of light irradiation; (**b**) Baseline corrected peak height at 470 nm absorbance peak from the top figure plotted against time of light irradiation. The slope of the fitted line is −0.041 absorbance/h.

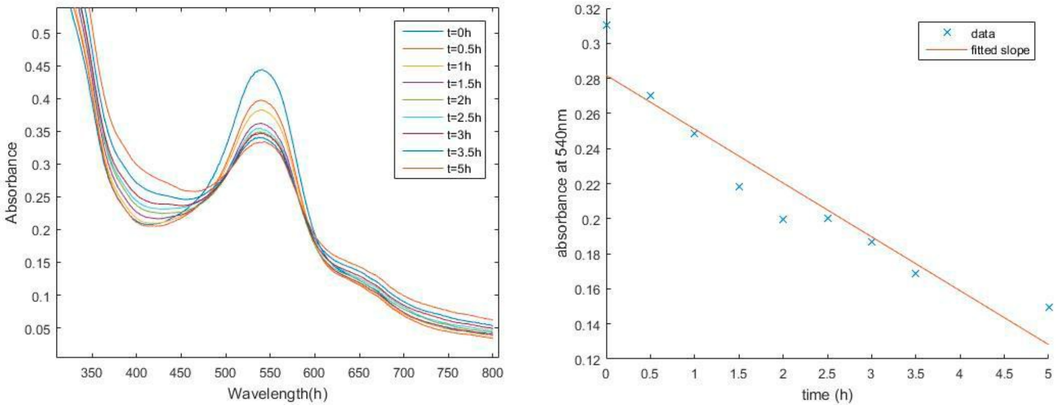

(**a**) (**b**)

Figure S6. Photostability test for NR patch: (**a**) Absorbance spectra of Laponite-Neutral red silk patch in 2 mL potassium phosphate buffer, 10 mM, pH 7 at different times of light irradiation; (**b**) Baseline corrected peak height at 540 nm absorbance peak from the top figure plotted against time of light irradiation. The slope of the fitted line is −0.031 absorbance/h.

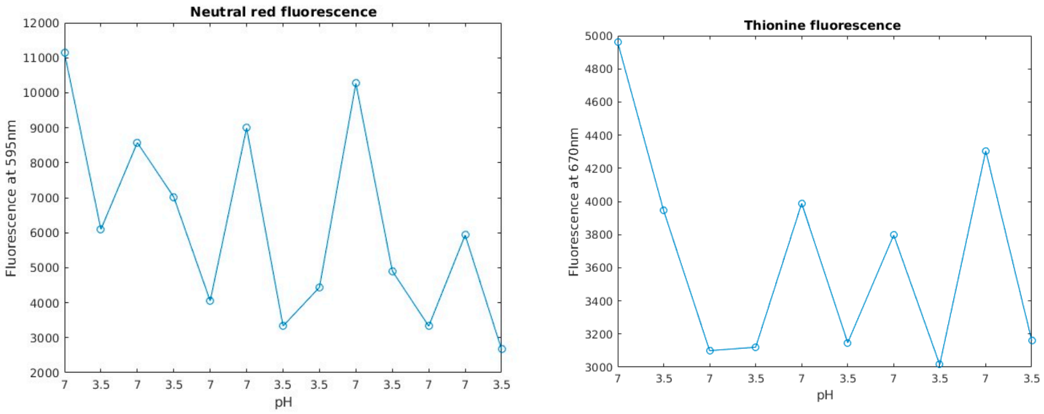

(**a**) (**b**)

Figure S7. Operational stability using fluorescence emission of NR (**a**) and Th (**b**). The *x*-axis is the successive run's pH values for both plots. Plotted are the maximum fluorescence at 595 nm for NR and 670 nm for Th.
